# Advances in the Comprehensive Tree Shrew Brain Atlas

**DOI:** 10.3390/biom16071027

**Published:** 2026-07-14

**Authors:** Wei Ma, Jinfen Zhang, Hongyu Liao, Tinghua Wang

**Affiliations:** 1 Institute of Neuroscience, Kunming Medical University, Kunming 650500, China; 2 Department of Neurology, The Second Affiliated Hospital of Kunming Medical University, Kunming 650101, China

**Keywords:** tree shrew brain atlas, neural progenitor cells, mouse brain atlas, non-human primate brain atlas, human brain atlas, single-cell, spatial transcriptomic brain atlas

## Abstract

The tree shrew is a small mammal characterized by a short gestation period, a relatively short lifespan, and low maintenance costs. It exhibits a close genetic relationship with primates and humans. The tree shrew possesses an eight-layered neocortex, limited cortical gyrification, an expanded subventricular zone, and sensory, visual, and motor cortices that resemble those of primates, along with comparable neurotransmitter systems, cognitive potential, and neural flexibility. These features render it a promising animal model for neurological disease research. A comprehensive understanding of its brain morphology, neural projections, neural circuits, and neuronal diversity is crucial both in terms of elucidating normal brain function and in order to establish this species as a reliable model in investigations of the mechanisms underlying neural injury and neurodegenerative diseases. Currently, studies on tree shrew brain development remain limited. This review presents a comprehensive summary of two-dimensional (2D) and three-dimensional (3D) comparative anatomical studies based on brain region localization, histological section staining, and MRI-derived brain imaging data in the tree shrew. It also analyzes the developmental characteristics of neural progenitor cells in this species. Furthermore, the review compares brain atlases of mice, non-human primates (NHPs), humans, and tree shrews generated using single-cell sequencing and spatial transcriptomic technologies. Finally, it outlines future research directions that emphasize the importance of integrating morphology and functional neuroimaging data with multi-omics data to construct a multimodal, four-dimensional (4D) brain development atlas of the tree shrew.

## 1. Introduction

### Tree Shrew: Biological Features and Molecular Evolutionary Classification

The tree shrew is a small mammal approximately the size of a rat, distinguished by a high brain-to-body mass ratio, large eyes, and remarkable mobility [1,2]. It reaches sexual maturity at around 5 months of age, has a short gestation period of 40–45 days, a relatively short lifespan, and lower maintenance costs compared to other mammals [1]. Tree shrews (*Tupaia belangeri*) belong to the order Scandentia, and 17 species are currently recognized according to the NCBI taxonomy database (https://www.ncbi.nlm.nih.gov/datasets/taxonomy/9394/, accessed on 3 May 2026). Considerable genetic disparities exist among different tree shrew species [3]. The high-quality genome sequence and annotation of the Chinese tree shrew (*Tupaia belangeri chinensis*) highlight its phylogenetic proximity to primates (see Supplemental Figure 1 in Savier et al., 2021) [4]. The findings suggest that tree shrews share a close genetic relationship with non-human primates (NHPs) and humans [5]. The annotated genome sequence of the Chinese tree shrew and its associated genome database (www.treeshrewdb.org) are publicly accessible [6]. Genome analyses reveal that tree shrews are genetically proximal to primates, with over 85% of their protein-coding genes conserved in humans. This genomic similarity extends from basic metabolism pathways to genes involved in brain development and immune response. Analyses of mitochondrial DNA, nuclear gene data, and whole-genome data consistently support a closer evolutionary relationship between tree shrews and primates [7]. Collectively, this research offers genetic evidence for the taxonomic classification and biological characteristics of tree shrews, further elucidating their evolutionary proximity to primates.

## 2. Advantages of Tree Shrews as Animal Models for Neurological Diseases

### 2.1. Neurological Disease Models in Tree Shrews

**Figure 1:**
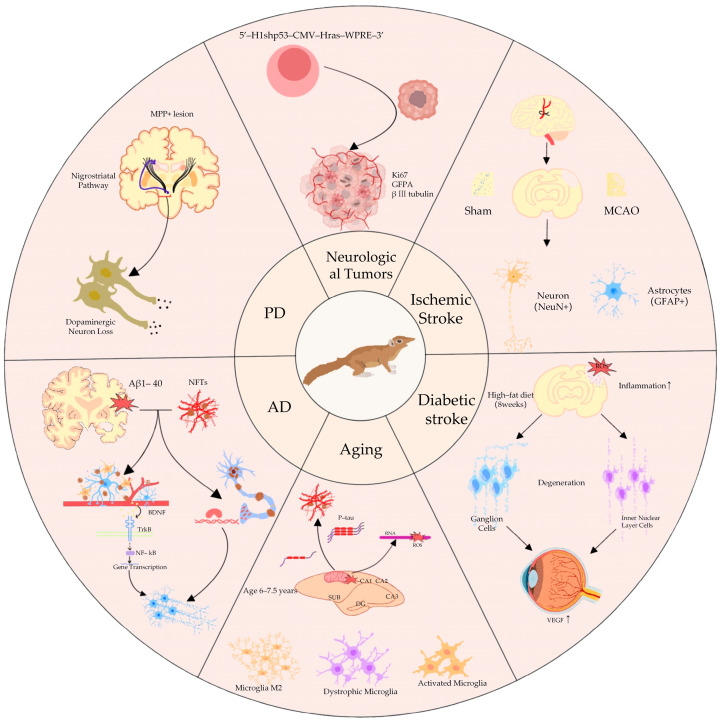
Tree shrews have emerged as promising animal models for studying aging, PD, AD, stroke, and neurological tumors.

Tree shrews exhibit both conserved features and unique characteristics in their nervous systems, which show greater similarity to those observed in NHPs than in rodents [3]. Consequently, tree shrews have emerged as a promising model for research on aging, neurodegenerative diseases (such as Alzheimer’s disease, AD; Parkinson’s disease, PD), stroke and neurological tumors [8,9,10,11,12,13,14,15,16,17] (Figure 1, Table 1).

### 2.2. Systematic Analysis of Brain Evolution and Function in Tree Shrews

During vertebrate brain evolution, subcortical structures remained relatively stable, whereas the cerebral cortex exhibited the greatest degree of variation [18]. The formation of the subventricular zone (SVZ) coincides with the emergence of the six-layered neocortex, and the expansion of basal progenitors—particularly basal radial glia (bRG)—is a key driver of neocortical expansion [19,20]. (1) In developing tree shrews, the neocortex differentiates into eight distinct areas with limited gyrification [21]. It features an expanded SVZ, abundant Pax6+ neural stem/progenitor cells within the SVZ, and a high proportion of bRG during peak upper-layer neurogenesis [22]. (2) Tree shrews possess a highly developed visual system [23]. Their visual cortex shows neuroanatomical and physiological homology with those of macaques and humans [24,25]. Layer 2/3 neurons display distinct ON/OFF pathways constrained by topographic organization, thereby supporting orderly columnar representations of stimulus orientation and visual space [26]. Cross-species single-cell RNA sequencing analyses reveal that tree shrew cone cells and subclasses are phylogenetically closest to human and macaque cell types [27]. (3) Tree shrews share subcortical similarities with NHPs. Their striatal chemical organization, including calcium-binding protein expression, aligns with that of both rodents and primates. Moreover, the distribution of dopaminergic projections and calcium-binding protein-labeled neurons in the striatum, along with basal ganglia circuit organization, preserves primate-conserved characteristics at histochemical and microcircuit levels, rather than exhibiting the rodent pattern, thereby indirectly corroborating structural similarity [28]. (4) Furthermore, they exhibit advanced neural functions, including cognitive learning and social behaviors, making them more comparable to primates than to traditional rodent models. Two studies demonstrate that tree shrews show greater cognitive potential than rats, based on assessments of spatial cognition, cognitive flexibility, and cooperative behavior using the pole board task and novel object recognition test [29,30], suggesting their suitability as a model for studying cognitive flexibility [31]. The tree shrew is a promising model for aging and AD research; unlike rodents, which differ by three residues, its Aβ42 peptide sequence matches that of humans [32]. Bilateral hippocampal lesions impair task performance, mirroring the cognitive decline observed in aging and AD models [33]. Furthermore, tree shrews acquire associative tasks more rapidly than spatial tasks, and their performance is notably affected by age and chronic stress [33].

### 2.3. Comparative Anatomy Study of Tree Shrew Brain Development

Few comparative anatomical studies have examined the development of the tree shrew brain. Expanding research in this area can improve our understanding of its neurodevelopment, provide valuable data for the use of tree shrews as models for brain disorders, and can potentially reduce reliance on NHPs. A comprehensive understanding of the morphological features, neural connectivity, functional circuits, and neuronal diversity of the tree shrew brain is crucial for elucidating normal brain function and for establishing tree shrews as valid and reliable animal models for the investigation of the behavioral, neurological, and molecular mechanisms underlying neural injury and neurodegenerative disorders. The morphology and physiology of the tree shrew’s nervous system have been studied, especially the anatomy and stereotactic localization of the brainstem, spinal cord, cerebral cortex, and thalamus [34,35,36,37,38,39,40]. Detailed descriptions of the localization and comparative anatomy of brain regions—including the amygdala, hypothalamus, thalamus, cerebellar nucleus, and piriform cortex—have also been provided [41,42]. The first systematic nomenclature and detailed mapping of the tree shrew brain were established using stained sections and 9.4 T MRI in the coronal, sagittal, and horizontal planes [43]. Nissl staining and acetylcholinesterase histochemistry were employed to identify the anatomical features of the cerebellum. Whole-brain afferent projections to the fifth cerebellar lobule and anterior paramedian lobule have been mapped [44]. These studies have identified more than 328 subregions and have defined the boundaries of key anatomical structures, including the olfactory bulb, basal ganglia, septal and preoptic regions, striatum, hypothalamus, thalamus, hippocampus, mesencephalon, metencephalon, and myelencephalon [45].

### 2.4. Neural Progenitor Cells (NPCs) in Tree Shrews Resemble Those in NHPs

Once neurogenesis begins, neuroepithelial cells and NPCs sequentially appear and contribute to the development of the cerebral cortex and its sulcal structure [46,47]. During this evolution, NPCs in the SVZ acquire key roles, such as generating the six cortical layers during brain expansion [19,20,48]. Most neocortical neurons originate from NPC populations in both the ventricular zone and SVZ. In animals with less developed sulci and gyri, such as rodents, intermediate progenitor cells (IPCs) dominate the SVZ and contribute to cortical thickening and surface expansion [22]. In contrast, NPCs in gyrencephalic animals, such as primates, can self-renew for up to five divisions [49,50]. Rodent IPCs mostly undergo neurogenic division, with only a small subset undergoing limited proliferative capacity before differentiating into neurons [51,52]. Notably, continuously dividing IPCs are rarely observed in NHPs [53]. Studies also show an expanded SVZ and primate-specific outer radial glial (oRG) cells in tree shrews. These oRG cells generate radial glial and intermediate progenitor cells, which subsequently differentiate into neurons. They express markers such as Sox2 and Pax6, supporting cortical development and the formation of sulci and gyri [22,54]. Studies comparing the primary visual cortex (V1) and prefrontal cortex (PFC) of primates have found unique specializations in pyramidal neurons (PNs) that are absent in mice. These morphological and physiological features likely contribute to the evolutionary expansion of the tree shrew cortex. This conclusion is supported by evidence including cytoarchitectonic boundaries, thalamic connectivity patterns, and layer II/III PN characteristics in V1 and PFC [40]. Research has also revealed key developmental features in the tree shrew (*Tupaia belangeri*) neocortex, including an expanded SVZ, a high abundance of Pax6+ NPCs in the SVZ, and increased basal radial glial (bRG) cells during peak upper-layer neurogenesis [55]. Additionally, tree shrew neural stem cells differ significantly from those of rats [56], suggesting a closer relationship to NHPs than to rodents.

In summary, tree shrews are closely related to primates, and their neurobiological features—including cortical organization, neurotransmitter systems, cognitive functions, motor control, visual processing, and stress responses—show homology with this order. Moreover, their neural progenitor cells and stress hormone secretion profiles resemble those of primates more closely than those of rodents, making tree shrews a valuable model for studying brain development and neuropathology.

## 3. Mouse, NHP, and Human Brain Atlases

The progression of brain atlas development in rodents, NHPs, and humans has advanced from traditional two-dimensional (2D) and three-dimensional (3D) anatomical and imaging atlases to contemporary high-resolution, pathway-level, single-cell spatial transcriptomic atlases at cellular and molecular scales. Traditionally, mouse brain atlases have relied upon 2D histological sections [57,58]. The Allen Institute’s Common Coordinate Framework version 3 (CCFv3) provides a 3D reference atlas that addresses the limitations of two-dimensional approaches and facilitates the integration of findings across diverse studies [59]. Recent advances in single-cell and spatial transcriptomics sequencing technologies have enabled characterization of brain cell types and subpopulations, elucidation of their spatial migration patterns, identification of region-specific genes, and revelation of cellular heterogeneity across brain regions throughout developmental stages from fetal to adult [60,61,62,63,64,65,66,67,68,69,70,71] (Table 2). Three-dimensional multimodal mouse brain atlases are invaluable for understanding neuronal diversity via comprehensive anatomical profiling.

**Figure 2:**
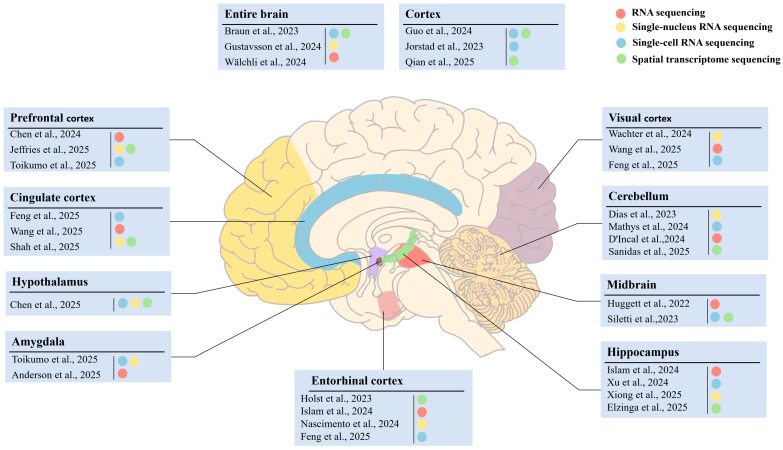
Single-cell RNA sequencing, single-nuclei RNA sequencing, and spatial transcriptomics studies reveal cellular and molecular heterogeneity in human brain development [77,78,79,82,83,84,85,86,87,88,89,90,91,92,93,94,95,96,97,98,99,100,101,102,103,104,105,106]. The revised schematic is adapted from Piwecka et al. (2023) [107] and updated and extended with post-2021 data.

NHPs possess developmental and neurological characteristics that exhibit a high degree of similarity to those of humans. Proteins associated with diseases serve as indicators of early fetal risks for neurodevelopmental disorders. Species-specific features and risk genes exhibiting mRNA-protein discrepancies have been identified through cross-species comparisons (involving humans, NHPs, and mice) and integrative analyses of single cells or proteomic–transcriptomic data in conjunction with anatomical mapping or neuroimaging (e.g., fMRI data). These include (Table 3): (i) a high-resolution transcriptional atlas of rhesus macaque brain development [72]; (ii) a stereotactic 3D whole-brain functional connectivity map of marmosets [73]; (iii) a cell type-resolved molecular atlas of the macaque cortex [74]; and (iv) a spatially resolved proteomic–transcriptomic atlas of cynomolgus macaques [75,76]. Recent studies have integrated single-cell and spatial transcriptomics to establish the spatiotemporal atlas of human embryonic and fetal brain development, revealing their molecular architectures and cellular diversities, and further elucidating the cellular and molecular heterogeneity associated with neurodevelopmental disorders, neural progenitor zones, and evolutionarily conserved transcription factors [77,78,79,80,81] (Table 4, Figure 2). However, research on brain development atlases in tree shrews remains scarce and has received limited attention.

## 4. Tree Shrew Brain Development Atlas

**Figure 3:**
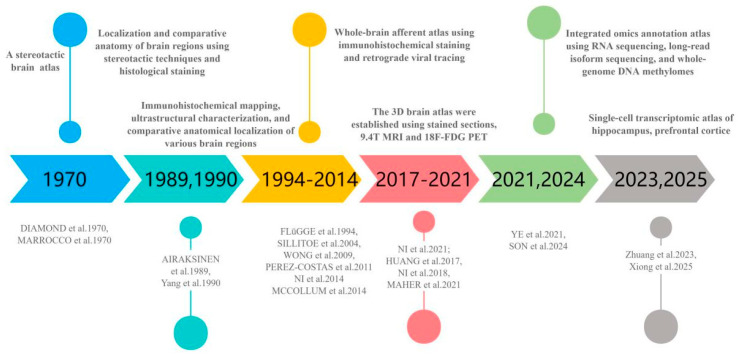
Chronological timeline of research advances in tree shrew brain atlases [18,21,28,35,36,37,38,41,42,43,44,45,93,109,110,111,112,113].

The development of the tree shrew brain’s developmental atlas has progressed from anatomical and neuroimaging maps to the recently constructed single-cell atlases [12,40,43,44,45,55,93,108,109] (Table 5, Figure 3).

### 4.1. Comparative Anatomy Atlas of the Tree Shrew Brain

One previous study established a stereotaxic coordinate system for the brain of Chinese tree shrews (*Tupaia belangeri chinensis*) and systematically mapped 328 brain subregions, clearly defining boundaries between key structures, including the olfactory bulb, basal ganglia, preoptic area, striatum, hypothalamus, thalamus, hippocampus, midbrain, hindbrain, and medulla [99].

### 4.2. Stereotaxic 18F-FDG PET and MRI Templates with a 3D Digital Atlas of the Tree Shrew Brain

A structural atlas of the tree shrew brain in the coronal, sagittal, and horizontal planes has been established through histological staining and stereotactic 9.4 T MRI. This atlas reveals the spatial localization of brain regions, the diversity of neural cell types, and the complex connectivity between the visual system and multiple cerebral areas [22,43].

### 4.3. Whole-Brain Afferent Atlas of the Tree Shrew Striatum

The distribution of whole-brain neuronal inputs projecting to the striatum in the tree shrew brain has been mapped using immunohistochemical staining and retrograde viral tracing. Structural and functional connections have been identified between the striatum and several brain regions, including the suprachiasmatic nucleus, caudate nucleus, putamen, nucleus accumbens [45], medial tegmental area [106], visual cortex [84], tectum, temporal cortex, striatum, hypothalamus, and optic tract. Neurotransmitter distribution in the tree shrew brain differs from that in rats, with each striatal subregion receiving convergent inputs from multiple upstream areas, such as the thalamus, cortex, and substantia nigra [45]. Despite advances in fluorescence staining, viral tracing, and MRI [43,106], mapping neural circuits in tree shrews remains challenging due to the scarcity of comprehensive, accurate, and single-cell-resolution connectivity data across brain regions.

### 4.4. Atlas of Tree Shrew Brain Development Through Integrative Analysis of Single-Cell Transcriptomics and Large-Scale Comparative Genomics and Epigenomics

Two-dimensional brain atlases are limited by their low spatial resolution and their inability to integrate multimodal data. Moreover, there is a lack of spatially resolved, dynamic 3D brain atlases with a single-cell resolution, which are essential for accurately defining brain structures, annotating cells, and tracking neural cell differentiation and migration during development. Recent advances have partially addressed these limitations by generating a cross-species (human, macaque, tree shrew, and mouse) single-cell transcriptomic atlas of the tree shrew hippocampus across postnatal developmental stages—infant, adult, and elderly—providing a comprehensive view of dynamic changes in neurogenic lineages, oligodendrocytes, microglia, and endothelial cell heterogeneity [93]. Cross-species comparisons reveal that the tree shrew transcriptome closely resembles that of macaques, highlighting its potential as a translational model for studying neurological disorders [105]. Previous analysis of human embryonic brain tissue identified 20 cell clusters across 14 major cell types, revealing primate-specific regulatory elements and spatiotemporal patterns of neural diversity during early brain development [114]. A recent study updated the annotation of the tree shrew genome using RNA-seq and ISO-seq data, identifying 3514 protein-coding genes and 50,576 lncRNAs. It revealed tissue-specific expression, alternative splicing, and orthologous genes across 11 species, as well as 144 unique gene families linked to infection response, such as IL6 and STT3B genes. Transcriptomic comparisons show that tree shrews are more similar to primates than to mice [93]. Research integrating methylome and transcriptome data from tree shrew prefrontal cortices has revealed a conserved role of DNA methylation in regulating XIST expression and indicates that CpG content affects sex-differential methylation at X-linked promoters, thereby advancing our understanding of mammalian sex chromosome evolution [82]. Another study presents a comprehensive single-cell transcriptomic atlas of the retina of tree shrews and other vertebrates, offering insights into neuronal diversity and evolution [88,90].

## 5. Prospects

### 5.1. Limitations and Technical Challenges of Using Tree Shrews as Animal Models for Neurological Diseases

This review also highlights the advantages and limitations of tree shrews as models for neurological diseases. (1) Since the completion of tree shrew genome sequencing [109,115], its genomic assembly has provided a crucial resource for the study of tree shrew genes and gene families. Specific gene sequences are accessible through public databases such as treeshrewDB [6,109] (www.treeshrewdb.org) and TupaiaBase [116] (http://tupaiabase.org). However, genetic backgrounds from different geographic regions may affect pathogen or disease susceptibility; therefore, the origin of the animals should be carefully considered when selecting experimental subjects. (2) The availability of immortalized tree shrew cell lines remains limited. To date, only immortalized cell lines derived from the intestinal epithelium, liver, and kidney of the Chinese tree shrew (*Tupaia belangeri chinensis*) have been reported [117,118,119]. No study has yet successfully established induced pluripotent stem cells or organoids in this species. (3) Tree shrew inbreeding is challenging. Currently, only the Kunming Institute of Zoology (Chinese Academy of Sciences) has established an inbred line; however, progress has been limited, with only a few lineages reaching the ninth generation (F9) [120]. (4) Tree shrew research faces challenges stemming from the lack of species-specific reagents and standardized toolkits—a limitation common to all non-model organisms [120].

### 5.2. Building a 4D Brain Development Atlas for Tree Shrews by Integrating Morphology, Functional Neuroimaging, and Multi-Omics Data

Current tree shrew brain atlases lack spatially resolved, single-cell data on neurogenesis across development. Existing methods—including staining, viral tracing [45], and MRI [43]—fail to generate a comprehensive, whole-brain, single-cell-resolution atlas of dynamic connectivity from embryogenesis to adulthood. Reconstructing a 4D brain development atlas for the tree shrew (Table 6)—capturing neural circuits, axonal projections, dynamic biological features, and single-cell-level connectivity and signaling—is essential to studying normal brain function and neurodegenerative disorders.

## 6. Conclusions

Our review summarizes recent advances in brain atlas research and available online databases (Supplemental Table S1) for mice, NHPs, humans, and tree shrews, and highlights the tree shrew’s utility as a model for human neurological disorders. Specifically, it highlights the development of the tree shrew brain atlas, including (1) a 2D whole-brain developmental atlas based on anatomical maps; (2) a stereotactic localization map; (3) stained-section maps in the coronal, sagittal, and horizontal planes using both the interaural and bregma reference systems; (4) MRI-based structural neuroimaging atlases; (5) whole-brain mapping of afferent and efferent connections; and (6) recently constructed dynamic 3D hippocampal atlases and two fetal whole-brain atlases generated using single-cell transcriptomic technology. It also discusses the advantages and limitations of tree shrews as models for neurological diseases, and the future prospects of the species as a model, such as the construction of a 4D tree shrew brain development atlas by integrating morphology, functional neuroimaging, and multi-omics data.

## Figures and Tables

**Table 1:** Tree shrew neurological disorder models.

Neurological Diseases	Phenotype or Cell Type	Potential Mechanism	References
Aging	1. M2 microglia activation. 2. Fewer ferritin+ oligodendrocytes	1. Oxidative stress causes tau hyperphosphorylation in neurons. 2. Iron overload generates ROS that damage RNA and contribute to microglial degeneration.	[8,9]
Neurodegenerative disease	A. AD 1. Cholinergic neuron injury 2. Astrocyte hyperactivation 3. Enhanced microglial inflammatory response 4. Tau protein hyperphosphorylation 5. Hippocampal atrophy, neuron apoptosis B. PD 1. Significant loss of dopaminergic neurons	A. AD 1. Aβ1-40 deposition induces oxidative stress and caspase pathway activation, leading to neuronal death. 2. Cholinergic dysfunction and BDNF/TrkB inhibition reduce neuronal survival and synaptic plasticity. 3. Rg1 modulates gut microbiota to activate gut–brain axis and alleviates neuroinflammation. 4. Rg1 inhibits tau phosphorylation and upregulates BDNF/TrkB signaling, promoting neuronal survival and synaptic function. B. PD 1. MPP+ inhibits mitochondrial complex I, selectively inducing dopaminergic neuron apoptosis and disrupting the nigrostriatal pathway. 2. Dopamine depletion causes motor control imbalance and PD-like symptoms.	[10,11,12,13]
Stroke	A. Ischemic stroke 1. NeuN+ neurons decrease 2. GFAP+ astrocytes decrease 3. Cell body atrophy 4. Nuclear pyknosis B. Diabetic stroke 1. Degeneration of retinal ganglion cells and inner nuclear layer cells	A. Stroke 1. Neuronal apoptosis. B. Diabetic stroke. 1. Oxidative stress. 2. Dysfunctional astrocytes impair blood–brain barrier maintenance. 3. Oxidative stress and inflammatory responses exacerbate neuronal injury. 4. VEGF-mediated angiogenesis.	[14,15,16]
Neurological tumors	1. Tumor cells express the markers Ki67, GFAP, and βIII-tubulin 2. The structure of neurons and glial cells is disrupted 3. High cell density, necrosis, and microvascular proliferation	1. H-Ras activation and p53 inactivation synergistically drive malignant transformation of neural cells. 2. Tree shrew p53 shares 93% homology with human p53. 3. Conserved post-translational modification sites.	[17]

**Table 2:** Developmental atlas of the mouse brain.

Method	Cell Type	Spatial Distribution	Gene	Highlight	Reference
1. Stereo-seq 2. DNA nanoball	1. Identified 8 types of excitatory glutamatergic neurons across cortical layers. 2. Four types of GABAergic interneurons with markers like Sst, Pvalb, Vip, Reln, and non-neuronal cells. 3. Clustered 281,377 cells into 25 major cell types.	Developing dorsal midbrain regions at E12.5, E14.5, and E16.5.	The top 1959 genes were identified.	1. Revealed the spatial cell-type heterogeneity of mouse embryonic tissues. 2. Mapped the spatiotemporal transcriptomic dynamics during mouse organogenesis. 3. Defined the spatiotemporal window of developmental disease vulnerability.	[60]
1. scRNA-seq 2. sci-Space 3. Immunohistochemical staining	1. Over 300 major cell types were identified. 2. Radial glial cells. 3. Chondrocytes. 4. Multiple neuronal subtypes.	1. Spinal cord of E14d mouse embryo. 2. Cortex of E14d mouse embryo.	Thousands of genes expressed in anatomical structures were identified.	1. Produced spatial atlas of gene expression at single-cell resolution. 2. Annotated diverse cell types and visualized cell type-specific spatial gene expression patterns. 3. Quantified the spatial position’s contribution to gene expression variation. 4. Analyzed cell developmental trajectories; reflected dynamic processes.	[61]
1. scRNA-seq 2. STARmap PLUS	1. Clustered and annotated 230 molecular cell types. 2. Identified 26 main cell types: 13 neuronal, 7 glial, 2 immune, and 4 vascular clusters.	Cerebral cortex, olfactory bulb, striatum, cerebellum, and brainstem.	Detected 1022 endogenous genes in 20 CNS tissue slices in situ.	1. Achieved single-cell resolution spatial mapping of molecular cell types. 2. Constructed molecular tissue region maps for precise anatomical delineation. 3. Created comprehensive molecular spatial cell type nomenclature and discovered new tissue structures and cell types. 4. Created a transcriptome-scale spatial atlas for the CNS and predicted gene expression patterns.	[62]
1. scRNA-seq 2. MERFISH 3. DAPI staining	Over 5000 transcriptionally distinct cell clusters were identified across 338 major cell types. Neurons have 315 subclasses and >5000 clusters; non-neuronal cells, 23 subclasses and 117 clusters.	Distributed in 11 adult mouse brain regions: olfactory areas, cortex, hippocampus, striatum, thalamus, hypothalamus, midbrain, hindbrain, cerebellum, fiber tracts, and ventricular systems.	Identified multiple neurotransmitter-related genes.	1. Imaged gene expression at single-cell resolution to show cell type distribution across the brain. 2. Integrated data for cell classification, type annotation, and transcriptome imputation. 3. Used CCFv3 registration to quantify cell type composition and organization in brain regions. 4. Analyzed cell–cell interactions to predict molecular bases and functional impacts.	[63]
1. scRNA-seq 2. MERFISH 3. Immunocytochemistry	Identified multiple cell types/subtypes, incl. 23 neurons: 8 BLA (2 excit, 6 inhib), P+T− neurons, and 3 astrocytes.	1. Basolateral. 2. Amygdala.	1. In total, 32 genes are regulated by long-term fear memory: Gpr88, Vip; 107 remote memory-associated DEGs: Plk2, NPAS4, and Trim.	1. Spatial transcriptomics mapped single-cell gene expression and located engram cells with neighbors. scRNA-seq identified memory-related genes and cell subtypes. 2. Viral vector and behavioral experiments verified specific cell and gene functions in memory formation. Integrative multi-omics revealed memory formation commonalities and differences across brain regions.	[64]
1. Single-neuron projection reconstruction 2. Spatial transcriptomics	Identified 43 single-neuron projection subtypes with distinct projection patterns.	Hippocampus (HIP).	N/A	1. Mapped single-neuron projections. 2. Analyzed the projection and organizational patterns of hippocampal neurons.	[65]
1. ANTsX software v2.0 toolkit 2. fMOST 3. MERFISH 4. AllenCCFv3	Divided cells into brain regions based on gene expression, such as cerebellum and cerebral cortex.	Cerebellum; cerebral cortex.	N/A	1. Provided precise spatial mapping and quantitative analysis tools for mouse brain research.	[66]
1. Integrate data from the BICCN community 2. Develop the CAR platform	1. Identified neuron types, including projection neuron classification. 2. Discovered key cellular features, such as three L5 neuron subtypes in primary somatosensory cortex.	Cortex, thalamus, and striatum.	N/A	1. Quantified neuronal morphology diversity and stereotypy for cell classification and regional segmentation. 2. Visualized brain region and connection structures to display brain organization.	[67]
1. scRNA-seq 2. snRNA-seq 3. ATAC-seq	1. Comprehensive description of brain cell types. 2. L4/5 IT neurons in the cortex.	1. Cerebral cortex. 2. Hippocampus. 3. Thalamus.		1. Provided a unified spatial framework for data positioning and integration by dividing the mouse brain into 23 subcortical structures, 41 fiber bundles, and 43 cortical regions.	[68]
1. MRI 2. Spatiotemporal RNA analysis technology	Identified 5 cell populations in lesion microenvironments.	Brain lesions: Perivascular and periventricular regions.	Discovery of significant enrichment in SASP-related genes, such as SERPINE1.	1. Used MRI pre-demyelination to detect early hypercellularity and locates future hotspots. 2. Monitored dominant cell type changes at different lesion development stages.	[69]
1. snRNA-seq 2. Stereo-seq 3. Spatial-ID algorithm	In total, 308 cell clusters were found—262 neuronal and 46 non-neuronal—featuring layer-/region-specific glutamatergic and GABAergic neurons in cerebral cortex, motor and neuromodulatory neurons in the brainstem, etc.	Divided brain into regions according to traditional anatomy and gene expression characteristics, such as dividing cortex into different layers and functional areas.	1. Detected 29,655 genes, including both coding and noncoding genes. 2. Dbh, Ddc, Tph2.	1. Developed the first whole-genome, single-cell-resolution spatial transcriptomic atlas of the entire mouse brain. 2. Mapped fine structural organization of brain regions, spatial distribution patterns of cell types, spatiotemporal dynamics of transcription factor regulation, and temporal–spatial expression profiles of lncRNAs.	[70]
1. 3D imaging experiments 2. TRISCO technique 3. Quantitative analysis by confocal microscopy	1. Neurons. 2. Astrocytes. 3. Microglia. 4. Oligodendrocytes and their precursors.	1. Brain; spinal cord.	Detected expression of multiple cell type marker genes, e.g., Pvalb, Mfge8, Sox10, and c-Fos.	1. Conducted whole-brain single-cell RNA imaging and spatial transcriptional analysis. 2. Presented expression patterns of multiple genes in the whole brain and detected neurotransmitter-related genes. 3. Evaluated neuronal functional activity by detecting c-Fos.	[71]

**Table 3:** Brain atlas of the non-human primate.

Technique/Method	Cell Type	Spatial Distribution	Gene	Highlight	Reference
1. LMD 2. DNA microarray technology 3. FISH	Identified 7 major categories: glial cells, neurons, neural progenitor cells, and intermediate progenitor cells.	Neocortex (medial prefrontal and visual cortical areas), hippocampus, striatum, and amygdala.	Identified the top 1000 genes with the largest increase and decrease in relative expression across all age pairs per structure.	Analyzed spatiotemporal gene expression changes in the developing primate brain to reveal molecular mechanisms and genes linked to neurodevelopmental disorders.	[72]
1. RS-fMRI 2. MRI	N/A	Cortical and subcortical gray matter regions of the brain.	N/A	Constructed the Marmoset FC Resource to provide data and tools for studying the marmoset brain’s functional organization.	[73]
1. RNA-seq 2. snRNA-seq 3. MRI	Identified six cell types linked to cortical imaging phenotypes in cynomolgus macaque: oligodendrocytes, microglia, astrocytes, OPCs, and excitatory and inhibitory neurons.	Cortical and subcortical regions of the brain.	Identified 971 protein-coding and 34 noncoding genes consistently associated with cortical thickness.	1. Analyzed gene expression and MRI cortical morphology links at whole-brain and cellular levels across species. 2. Identified co-expression modules and gene associations.	[75]
1. snRNA-seq 2. Stereo-seq 3. Immunohistochemistry	1. Identified 264 transcriptome-defined cortical cell types, incl. glutamatergic, GABAergic, non-neuronal, etc. 2. L4 glutamatergic neurons.	Divided the cerebral cortex into 143 regions, such as the frontal lobe and parietal lobe.	1. Researched multiple genes: cell markers, neurotransmitters, and receptors. 2. Highly expressed in primate-specific L4 cell types (e.g., FOXP2, EPHA3, DCC).	1. Analyzed cortical cell types and spatial distribution in macaques, exploring their relationship with cortical hierarchy. 2. Discovered primate-specific cell types and provided a data-based foundation for studying brain evolution, development, aging, and pathogenesis.	[74]
1. 4D-proteomics technology 2. Immunohistochemistry technique	1. Purkinje’s cell. 2. Granulosa cell. 3. Neural stem cell.	Divided the brain into 18 regions, e.g., neocortex, cerebellum, striatum, and hippocampus.	Researched genes related to brain development (e.g., MCM2-7, APP) and neurological disorders (e.g., STXBP1).	1. Revealed spatiotemporal changes in cynomolgus monkey brain protein expression from fetal to neonatal stages. 2. Analyzed relationships between protein expression changes, brain development and diseases, and conducted cross-species comparisons.	[76]

**Table 4:** Human brain atlas.

Technique/Method	Cell Type	Spatial Distribution	Gene	Highlight	Reference
1. scRNA–seq 2. snRNA–seq 3. Spatial transcriptomics technology	1. Radial glial cells (RGCs). 2. Intermediate progenitor cells (IPCs).	The hypothalamus.	Multiple genes involved in neural pattern formation, neuronal differentiation, and neural regulation.	1. Analyzed the developmental and evolutionary characteristics of hypothalamic cells. 2. Mapped the spatial pattern of hypothalamic progenitor cells and reconstructed the neurogenic lineage.	[77]
1. scRNA-seq 2. Spatial transcriptomics technology	1. Divided cells into 5 clusters: RGC, IPC, Neurons (Ns), INs, and MG. 2. Annotated 7 clusters in cortex at GW18: RGCs, IPCs, neurons, INs, OPCs, MG, and ECs. 3. Subplate neurons (SPNs) 4. Intermediate progenitor cells.	Human fetal cerebral cortices from GW10 to GW25.	1. Screened and compared 690 DEGs. 2. Detected high expression of 124 SPN marker genes at different gestational weeks. 3. Observed expression patterns of PAX6, EOMES, NEURAD2, and NR4A2 in cerebral cortex.	1. Revealed molecular characteristics, origin, and developmental trajectories of subplate neurons in human fetal cerebral cortex, and clarified their mechanism in brain development. 2. Explored using spatial transcriptomics and single-cell RNA sequencing from multiple perspectives.	[78]
1. ATAC-seq 2. RNA-seq 3. MERFISH 4. Single-cell profiling	1. Identified a tripotential intermediate progenitor subtype. 2. Identified 5 classes, 11 subclasses, and 33 high-fidelity cell types. 3. Identified 10 macroglia lineage cell types, including three RGs, IPC-Glia, and others associated with astrocyte or oligodendrocyte lineages.	Distributed in the human neocortex, including PFC and primary visual cortex (V1).	1. Designed a 300-gene panel for the main cell types in the developing cortex. 2. Identified 582 eRegulons: 385 activators and 197 repressors. 3. Identified 1908 differentially expressed genes between V1-specific and common EN-L4-ITs.	1. Built a multi-omic atlas of human neocortex development. 2. Revealed molecular and cellular dynamics and created a disease risk map to understand brain development and disease mechanisms.	[79]
1. scStereo-seq 2. scRNA-seq	1. Identified 19 main cell types and multiple cell subtypes. 2. Radial glial (RG) cells.	Multiple regions of the brain, including Cor, Sp, diencephalon, Mid, and Cere.	Researched multiple genes, including cell marker, transcription factor, and extracellular matrix-related genes.	1. Developed a spatiotemporal atlas of multiple human brain regions to reveal regional specification mechanisms during brain development. 2. Identified commonalities of GABAergic neuron subtypes across brain regions and demonstrated the impact of OPC-GABAergic neuron interactions on neuronal development and regional norms.	[80]
1. scRNA-seq 2. Fluorescence-activated cell sorting(FACS)	1. Identified ten NSPC types, e.g., radial glia, neuron precursors, oligodendrocyte precursors, and astrocyte lineage cells. 2. Bipotent glial progenitor cell (GPC).	1. Divided human fetal brain into 13 distinct regions, e.g., frontal lobe, motor cortex, and somatosensory cortex. 2. GW16–20 human brains.	Cell differentiation and fate determination (e.g., SOX2, PAX3).	1. Purified and functionally identified different NSPC types. 2. Supported study of self-renewal, differentiation potential, and lineage relationships of cells in human neural development.	[81]

**Table 5:** Some representative tree shrew brain atlases.

Technique/Method	Cell Type	Spatial Distribution	Gene	Highlight	Reference
Genome seq.	N/A	N/A	The genes of multiple viruses were studied to determine their classification and evolutionary relationships.	Detected and analyzed viruses in tree shrew samples for genomic characteristics and evolutionary relationships.	[108]
1. Immunocytochemistry 2. Image acquisition and analysis	1. Identified multiple types of neural stem and NPCs, such as Aps and BPs. 2. Basal radial glial cell (bRG).	Developing neocortex.	Multiple genes, including markers such as Pax6 and Tbr2.	Revealed neural progenitor cell characteristics, distribution, and quantitative changes in tree shrew neocortex development, and compared them with those of other species.	[55]
1. Whole-cell patch-clamp recording 2. Immunohistochemistry	Pyramidal neurons (PNs).	1. Primary visual (V1). 2. Dorsal prefrontal cortex (DFC). 3. Medial prefrontal (MF).	N/A	Revealed morpho-physiological differences in pyramidal neurons across tree shrew brain regions using multiple techniques.	[40]
1. Immunohistochemistry 2. EdU and BrdU dual-pulse labeling	1. Top progenitor cells (APs). 2. Basal progenitor cells (BPs). 3. Intermediate progenitor cells (IPCs).	Neocortex.	Cell marker genes (such as Sox2 and Pax6)	Studied neural progenitor cells in tree shrew neocortex to reveal roles in brain development and evolution.	[12]
1. Immunohistochemistry 2. Retrograde tracer Fluoro-Gold (FG)	Neurons expressing PV, NOS, CR, and TH.	Striatum (including caudate nucleus (Cd), putamen (Pu), internal capsule (ic), accumbens nucleus (Acb)).	PV, NOS, CR, and TH genes.	1. Clarified cellular and chemical structure of tree shrew striatum. 2. Mapped whole-brain inputs to striatal sub-regions; quantified cell density and fiber intensity. 3. Analyzed striatal protein distribution differences between tree shrews and rats.	[45]
1. PET/CT and MRI 2. Reconstruction of the digital atlas	N/A	Digitization and regional division of 628 brain structures, including olfactory bulb and striatum.	N/A	Constructed a digital atlas of and templates for the tree shrew brain to enable image normalization and statistical analysis, supporting neuroimaging research.	[43]
1. RNA-seq 2. ISO-seq	N/A	Small intestine, liver, heart, kidney, spleen, ovary, brain, and testis.	1. Obtained 27.1 k coding genes (incl. 3.5 k newly annotated) and 50.6 k lncRNA genes. 2. Identified 144 tree shrew-specific gene families (incl. IL6 and STT3B). 3. Detected 272.8 k genes across 11 mammals.	1. Identified 144 tree shrew-specific gene families. 2. Identified high-quality tree shrew transcripts. 3. Conducted tissue expression comparison across human, rhesus monkey, tree shrew, and mouse models; indicated orthologous relationships of tree shrew genes with those of primates.	[109]
1. Nissl staining 2. AChE histochemistry 3. Immunohistochemistry	Identified cerebellar cell types, e.g., Purkinje and granule cells. Cerebellar Purkinje cells.	1. Divided cerebellum into 32 subregions and 10 fissures; fissures divide vermis into 10 lobules. 2. Purkinje cell layer (Pk) of cerebellar cortex.	Cerebellum and whole-brain projections to the cerebellar cortex.	1. Described tree shrew cerebellum anatomy, neurochemistry, and afferent projections. 2. Created a cerebellum atlas.	[44]
1. snRNA-seq 2. Immunohistochemistry	1. Identified 7 major categories and 42 cell populations, including neurogenic lineage, oligodendrocytes, microglia, and endothelial cells. 2. Identified a unique class of tree shrew-specific neural stem cell.	Hippocampal tissue.	1. Genes linked to nervous system disorders and aging in cell types from humans, macaques, mice, and TS. 2. Divided genes into four clusters.	1. Analyzed hippocampal cell composition, molecular features, and interactions in tree shrews. 2. Constructed a single-cell transcriptomic atlas; compared it with those of humans, macaques, and mice; and studied aging characteristics.	[93]

**Table 6:** Future study plans and the methodology for the tree shrew development brain atlas.

Embryonic Developmental Stages	Morphology Atlas+ Computer Reconstruction	Functional Imaging Atlas	Spatiotemporal Atlas	Single-Cell Atlas	Cross Species Analysis
Early (Prior to the E30)	H&E staining. Nissl staining. Immunofluorescence staining networks of neuronal and glial cells.	PEC-CT. fMRI.	Spatial transcriptomics sequencing. Spatial proteome sequencing.	Single-cell transcriptome sequencing. Single-cell proteome sequencing.	Download the single-spatial transcriptomics data of mice, NHPs, humans.

## Data Availability

No new data were created or analyzed in this study. Data sharing is not applicable to this article.

## Supplementary Materials

The following supporting information can be downloaded at https://www.mdpi.com/article/10.3390/biom16071027/s1: Table S1: Brain atlases databases for mouse, non-human primate, human and tree shrew.

## Abbreviations

2D	two-dimensional
3D	three-dimensional
4D	four-dimensional
NHPs	non-human primates
AD	Alzheimer’s disease
PD	Parkinson’s disease
MRI	magnetic resonance imaging
NPCs	Neural progenitor cells
SVZ	subventricular zone
IPCs	intermediate progenitor cells
oRG	outer radial glial
bRG	basal radial glia
V1	primary visual cortex
PFC	prefrontal cortex
PNs	pyramidal neurons
CCFv3	Common Coordinate Framework version 3
