# Supplementary material for: Advances in the Comprehensive Tree Shrew Brain Atlas
Source: Biomolecules. 2026 Jul 14;16(7):1027. doi: 10.3390/biom16071027 (PMC13407228; doi:10.3390/biom16071027)
Supplement: Supplementary file 1 [file biomolecules-16-01027-s001.zip › biomolecules-4340140-supplementary.pdf]

Table S1. Brain atlases databases for mouse, non-human primate, human and tree shrew.

Species	Database	Main contents
Mouse	https://mouse.brain-map.org/ (The URL from the Allen Institute for Brain Science)	1.Reference Atlas, Version 1 (2008).<br>2.Reference Atlas, Version 2 (2011).<br>3.Mouse CCF, Reference Atlas, Version 3 (2015).<br>4. The Allen Brain Cell Atlas.<br>5.A high-resolution transcriptomic and spatial cell-type atlas.<br>6. Allen Developing Mouse Brain Atlas: Allen Developing Mouse Brain Atlas: Allen Developing Mouse Brain Atlas: In Situ Hybridization (ISH) Data, Developmental Anatomic Gene Expression Atlas (AGEA).<br>......
non-human primates	https://brain-map.org/atlases# non-human-primate (The URL from the Allen Institute for Brain Science)(The URL from the Allen Institute for Brain Science)	1.Multi-modal information about non-human primate cell types.<br>2.Adult & Developing NHP Atlas
Human	https://human.brain-map.org/ (The URL from the Allen Institute	1.In Situ Hybridization in the Human Brain Atlas.<br>2.Human Brain Atlas: Microarray Data.

	for Brain Science)	3.Human Brain Atlas: MRI.<br>4.BrainSpan Atlas of the Developing Human Brain: Brain Span bulk RNA-Seq data, BrainSpan-Developmental Transcriptome, BrainSpan-In Situ Hybridization Data, BrainSpan - Prenatal LMD Microarray.<br>......
Tree shrews	http://www.treeshrewbd.org.<br>http://www.fil.ion.ucl.ac.uk/sp<br>(Not open access) [43, 113, 115].	1.Genomic data<br>2.Mitochondrial genome data.<br>3.Stereotaxic 18F-FDG PET and MRI templates with three-dimensional digital atlas.
